# The role of serum adropin in determining the clinical outcomes of patients with traumatic brain injury: a case-control study

**DOI:** 10.1590/1806-9282.20240275

**Published:** 2024-07-19

**Authors:** Özlem Tataroğlu, Oya Güven, İlhami Demirel, Özgür Söğüt, Mehmet Yiğit, Olgun Demir, Esma Özdemir Anayurt

**Affiliations:** 1 University of Health Sciences, Dr. Lütfi Kırdar City Hospital, Department of Emergency Medicine – İstanbul, Turkey.; 2 Kırklareli University, School of Medicine, Department of Emergency Medicine – Kırklareli, Turkey.; 3 University of Health Sciences, Haseki Research and Training Hospital, Department of Emergency Medicine – İstanbul, Turkey.; 4 University of Health Sciences, Haseki Research and Training Hospital, Department of Biochemistry – İstanbul, Turkey.

**Keywords:** Adropin protein, Brain injury, Emergency room

## Abstract

**OBJECTIVE:**

It has been determined that adropin has a role in tissue healing. This study aimed to determine the effects of head trauma on the tissues and blood levels of patients admitted to the emergency department.

**METHODS:**

The study group was divided into two to compare the adropin level in healthy individuals and patients with head trauma. Blood tests from patients and healthy volunteers were compared using the adropin kit. Adropin levels, Glasgow Coma Scale, and revised scores of trauma patients were recorded and analyzed.

**RESULTS:**

All patients in the trauma group had significantly higher adropin levels than the control group. Among these patients, the adropin level of the discharged patients was higher than the others. In addition, patients with high Glasgow Coma Scale and normal blood pressure were found to have higher adropin levels than the others.

**CONCLUSION:**

Although adropin cannot make a sharp distinction in determining the prognosis, the increase in its level in trauma patients shows that it triggers a protective mechanism.

## INTRODUCTION

Adropin is a peptide hormone encoded by the Energy Homeostasis Associated (Enho) gene located on chromosome 9 in the central nervous system (CNS), heart, kidney, liver, pancreas, and human umbilical vein^1^. Studies show that it modulates endothelial nitric oxide synthase (eNOS) expression and provides cytoprotective and vasculoprotective effects^2,3^. Studies have shown that the level of adropin is low during acute ischemia, increases gradually, and can be an alternative to troponin, especially in the follow-up of myocardial infarction^4^. It is thought that diseases such as diabetes and vasculitis may develop in their absence^5,6^.

Studies at the molecular and cellular levels in recent years have shown that adropin, as a biochemical parameter, plays an essential role in the pathogenesis and progression of CNS diseases (stroke, bipolar disorder, schizophrenia, schizophrenia, bipolar disorder, Alzheimer's, Parkinson's, and Huntington's diseases) (one). In animal studies, it has been shown that adropin regulates locomotor activity and plays a role in the development of the cerebellum. It does this through the neuronal recognition molecule 3 (NB3/Notch) cascade as a membrane-bound protein^7,8^. In ischemia, it activates vascular endothelial growth factor receptor 2 (VEGFR2), and apoptosis occurs due to cascade activation. Thus, the width of the ischemia area in the brain increases in direct proportion to the level of adropin in the blood^9^.

Head trauma is a type of injury that can be fatal. Considering the intensity of the emergency department (ED), new strategies are needed to facilitate patient follow-up and predict mortality, especially in the ED, which is the first place of admission for head trauma patients. This study indicated that adropin, whose various effects were discovered and popularized daily, could also be used in the follow-up of trauma patients. In addition, it will be discussed whether the findings obtained in the study affect the determination of medical priority in this patient group.

## METHODS

### Ethical approval

This study was approved by the Local Ethics Committee (2018-14/8.5.2018). Written and verbal consent was obtained from all patients or their relatives who participated in the study.

### Study type

The is a single-center, prospective, randomized, controlled study.

### Study sample

All male and female patients over 18 years who came to the ED due to isolated head trauma between June 1 and August 30, 2018, and healthy volunteers without a history of trauma or any other complaint were included in the study.

Patients with multiple traumas who did not want to consent and were under 18 years old or the healthy population were excluded from the study.

### Data collection

Written and verbal consent was obtained from the patients and healthy volunteers who volunteered to participate in the study, and they were divided into two groups: patient and control. The patients were stabilized hemodynamically, and 3-5 mL of blood samples were taken into the biochemistry tube at any time from healthy adult individuals in the control group in the early post-traumatic period (within the 6 h). Blood samples were centrifuged at 3,000 rpm for 5 min and kept at 80°C according to the manufacturer's protocol. Serum human adropin was determined using commercial enzyme-linked immunosorbent assay (ELISA) kits (BT-LAB, China). Serum samples were not diluted with those kits, and the standards were serially diluted from starting concentrations of 640 ng/L down to 40 ng/L for adropin in the sample diluent supplied with the kit. The intra- and inter-assay coefficient of variation for the assays is<10%.

Age, gender, vital signs, and trauma scores of all patients and volunteers in the study, as well as trauma scores [Glasgow Coma Scale (GCS) and Revised Trauma Score (RTS)], trauma mechanism, brain computed tomography (CT) findings, serum adropin levels, and clinical results (exitus, hospitalization, and discharge) were noted. Patients with trauma were divided into three groups: I (GCS 3-8), II (GCS 9-13), and III (GCS 14-15), according to GCS scoring, and serum adropin levels were compared between the groups.

### Sample size and statistical method

In the descriptive statistics of the data, mean, standard deviation, median minimum, maximum, frequency, and ratio values were used. The distribution of variables was measured using the Kolmogorov-Smirnov test. The Kruskal-Wallis and Mann-Whitney U tests were used to analyze independent quantitative data. The chi-square test was used to analyze independent qualitative data, and the Fisher's test was used when the chi-square test conditions were not met. The effect level and cutoff value were investigated with the ROC curve. The SPSS 28.0 program was used in the analysis.

## RESULTS

In the study, 58 (32.2%) were female, and 122 (67.8%) were male, of which 118 (65.5%) were in the patient group and 62 (34.5%) were in the control group. The mean age was 46.3±18.4 years in the control group and 44.8±18.8 years in the patient group (p>0.05). Adropin levels increased approximately two times in the patient group compared with the control group (p<0.05). A total of 26 patients (22.0%) were hospitalized, and 4 (15.3%) of these patients died (Table 1).

**Table 1:** Demographic data and adropin levels of the groups in the study.

Mean± SD/n (%)					
		Healthy group	Discharged	Inpatient	p
Age		46.3±18.4	45.4±19.9	42.5±14.1	0.748^k^
Gender					
	F	24-38.7%	30-32.6%	4-15.4%	
	M	38-61.3%	62-67.4%	22-84.6%	0.101x^2^
	Adropin Level	148.1±182.0	222.5±292.4	180.8±217.2	**0.001** ^k^
	Adropin ≤80	36-58.1%	24-26.1%	6-23.1%	
	>80	26-41.9%	68-73.9%	20-76.9%	**0.000x** ^2^
	GCS I	–	–	9-34.6%	
	II	–	–	11-42.3%	**0.000x** ^2^
	III	63-100%	92-100%	6-23.1%	

GCS: Glasgow Coma Scale; k: Kruskal-Wallis (Mann-Whitney U test); X^2^: chi-square test.

Significant values (P<0.05) are marked in bold.

**Figure 1:**
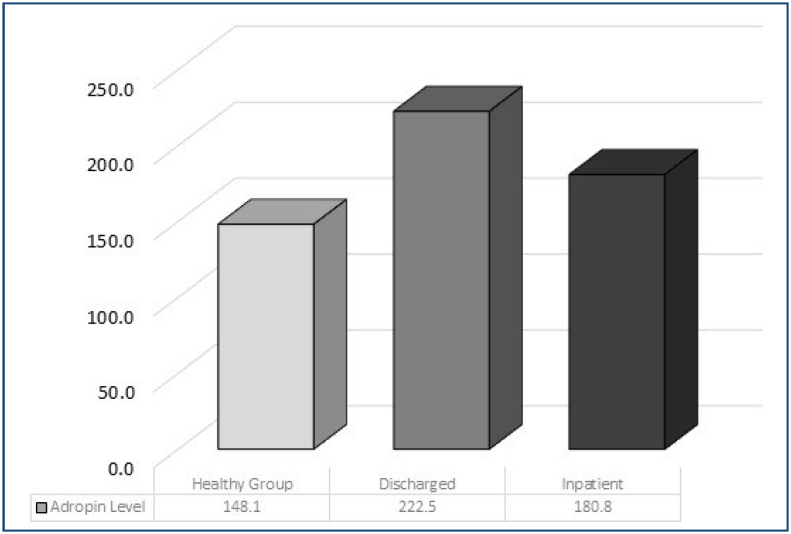
Comparison of adropin levels between groups.

All patients in the trauma group had significantly higher adropin levels than the control group. Among these patients, the discharged patients' adropin level was higher than the others (Figure 1).

The adropin cutoff level in the patient group in the study was accepted as 80 ng/l and divided into two groups. No pathology was detected in the CT results of 92 patients (77.9%). Subarachnoid bleeding was observed at the highest rate (53.8%) in patients with pathological CT results. Adropin level was high in most patients with normal or abnormal CT results. In addition, patients with high GCS and normal blood pressure had higher adropin levels than the others (Table 2).

**Table 2:** Results in groups according to adropin levels.

		Adropin ≤ 80				Adropin > 80				p	
		Mean±SDn (%)			Median	Mean±SD n (%)			Median		
BT*	Normal	21		80.8%		71		77.2%		0.696	X^2^
	Abnormal	5		19.2%		21		22.8%			
Epidural		1		3.8%		4		4.3%		1.000	X^2^
Intraparenchymal hemorrhage		0		0.0%		1		1.1%		1.000	X^2^
Subdural		1		3.8%		5		5.4%		1.000	X^2^
SAH		4		15.4%		10		10.9%		0.530	X^2^
Contusion		2		7.7%		3		3.3%		0.304	X^2^
Linear fracture		3		11.5%		5		5.4%		0.372	X^2^
Deplete fracture		0		0.0%		1		1.1%		1.000	X^2^
Others		0		0.0%		2		2.2%		1.000	X^2^
SBP		131.4	±	18.5	133.0	124.1	±	16.0	122.0	***0.018***	x^m^
DBP		80.0	±	11.9	80.0	73.1	±	10.4	70.0	***0.003***	x^m^
RTS		11.9	±	0.4	12.0	11.8	±	0.7	12.0	0.699	x^m^

* More than one CT is found in the same patient.

x^m^: Mann-Whitney U test; X^2^: chi-square test (Fisher's test). CT: computed tomography; SAH: subarachnoid hemorrhage; GCS: Glasgow Coma Scale; Others: diffuse axonal injury, extra-axial hemorrhage; SBP: systolic blood pressure; DBP: diastolic blood pressure; RTS: Revised Trauma Score.

Significant values (P<0.05) are marked in italics.

## DISCUSSION

Most of the studies on adropin are on its effects on endocrine or neurological diseases. This study showed that the level of adropin increased in trauma patients.

There was no difference in the mean age of the patients included in the study and healthy volunteers (p>0.05). Thus, the early inflammatory response after trauma, which is observed more rapidly in the elderly population than in young people, was not likely to trigger secondary brain damage^10^. Accordingly, the significantly higher adropin level in the patient group from the two physiologically homogeneous groups compared with the other group is strongly evident that adropin can be used in post-traumatic follow-up.

Cell necrosis that develops after hypoxia-induced vasospasm is held responsible for the pathophysiology of brain injury. This situation is like ischemic stroke, but in stroke, cerebral blood flow must decrease to deficient levels (5–8.5 mL/100 mL/min). In traumatic brain injury, necrosis develops after higher blood flow (15 mL/100 mL/min), which explains the higher adropin level observed in ischemic stroke in a shorter time^9,11^. In this study, the fact that the adropin level in the patient group was higher than the healthy group indicates that the adropin level increased with the hypoxic damage caused by the trauma. In addition, although the level of adropin increases in the acute period of traumatic brain injury, it can be predicted to continue to increase during the post-traumatic tissue edema, degeneration, and regeneration process in long-term follow-up for patients for recovery.

Axonal damage after head trauma cannot be detected on imaging. The patient is followed up when neurological symptoms or bleeding are seen on CT. Apart from these, it may not show any symptoms in the acute period. The ED follow-up of patients in this group may be affected. A study found that endothelial proliferation and angiogenesis increased in mice injected with adropin after vascular injury^3^. In this study, the result was normal in most patients who underwent CT, but the adropin level was high, and the adropin level was high in most patients with pathology on CT. This demonstrates the promoting role of adropin in healing tissue damage regardless of trauma severity.

Studies have shown that adropin level is decreased in hypertensive patients compared with normotensive patients. It has been stated that these patients are prone to risks that may develop after endothelial dysfunction, and the endothelial protective effect of adropin has been emphasized^12,13^. In this study, adropin level was higher in normotensive patients than in hypertensive patients.

## CONCLUSION

Most of the patients in the study were discharged because of high GCS levels, and most had high adropin levels. According to this, although adropin cannot make a sharp distinction in determining the prognosis, the increase in its level in trauma patients shows that it triggers a protective mechanism. In addition, the clinician may consider discharging the patient with a markedly high (200 ng/L) adropin level.

This study observed that the adropin level was higher in the patient group with high GCS. We think that in this patient group with a good prognosis and discharged from the ED, the adropin level can be taken at regular intervals during the outpatient control, and the tissue healing process after trauma and the complications that may develop can be followed up.

### Limitations

There are some limitations in the study. Only the adropin level taken in the ED follow-up was measured. The adropin levels in the hospitalized patients' service or intensive care follow-up could not be determined. According to the result of this study, although it is predicted that the blood level will increase more significantly in long-term patient follow-up, long-term studies are needed to determine the time to return to the average level.

## References

[1] Aydin S (2014). Three new players in energy regulation: preptin, adropin and irisin. Peptides.

[2] Shahjouei S, Ansari S, Pourmotabbed T, Zand R (2016). Potential roles of adropin in central nervous system: review of current literature. Front Mol Biosci.

[3] Lovren F, Pan Y, Quan A, Singh KK, Shukla PC, Gupta M (2010). Adropin is a novel regulator of endothelial function. Circulation.

[4] Aydin S, Kuloglu T, Aydin S, Kalayci M, Yilmaz M, Çakmak T (2014). Elevated adropin: a candidate diagnostic marker for myocardial infarction in conjunction with troponin-I. Peptides.

[5] Celik E, Yilmaz E, Celik O, Ulas M, Turkcuoglu I, Karaer A (2013). Maternal and fetal adropin levels in gestational diabetes mellitus. J Perinat Med.

[6] Gao F, Fang J, Chen F, Wang C, Chen S, Zhang S (2016). Enho mutations causing low adropin: a possible pathomechanism of MPO-ANCA associated lung injury. EBioMedicine.

[7] Wong CM, Wang Y, Lee JT, Huang Z, Wu D, Xu A (2014). Adropin is a brain membrane-bound protein regulating physical activity via the NB-3/Notch signaling pathway in mice. J Biol Chem.

[8] Chen H, Qu Y, Tang B, Xiong T, Mu D (2012). Role of mammalian target of rapamycin in hypoxic or ischemic brain injury: potential neuroprotection and limitations. Rev Neurosci.

[9] Altintas O, Kumas M, Altintas MO (2016). Neuroprotective effect of ischemic preconditioning via modulating the expression of adropin and oxidative markers against transient cerebral ischemia in diabetic rats. Peptides.

[10] Timaru-Kast R, Luh C, Gotthardt P, Huang C, Schäfer MK, Engelhard K (2012). Influence of age on brain edema formation, secondary brain damage and inflammatory response after brain trauma in mice. PLoS One.

[11] Cunningham AS, Salvador R, Coles JP, Chatfield DA, Bradley PG, Johnston AJ (2005). Physiological thresholds for irreversible tissue damage in contusional regions following traumatic brain injury. Brain.

[12] Gulen B, Eken C, Kucukdagli OT, Serinken M, Kocyigit A, Kılıc E (2016). Adropin levels and target organ damage secondary to high blood pressure in the ED. Am J Emerg Med.

[13] Gu X, Li H, Zhu X, Gu H, Chen J, Wang L (2015). Inverse correlation between plasma adropin and ET-1 levels in essential hypertension: a cross-sectional study. Medicine (Baltimore).

